# Cerebellar gene expression profiles of mouse models for Rett syndrome reveal novel MeCP2 targets

**DOI:** 10.1186/1471-2350-8-36

**Published:** 2007-06-20

**Authors:** ChaRandle Jordan, Hong Hua Li, Helen C Kwan, Uta Francke

**Affiliations:** 1 Department of Genetics, Stanford University School of Medicine, Stanford CA 94305-5323, USA

## Abstract

**Background:**

MeCP2, methyl-CpG-binding protein 2, binds to methylated cytosines at CpG dinucleotides, as well as to unmethylated DNA, and affects chromatin condensation. *MECP2 *mutations in females lead to Rett syndrome, a neurological disorder characterized by developmental stagnation and regression, loss of purposeful hand movements and speech, stereotypic hand movements, deceleration of brain growth, autonomic dysfunction and seizures. Most mutations occur *de novo *during spermatogenesis. Located at Xq28, *MECP2 *is subject to X inactivation, and affected females are mosaic. Rare hemizygous males suffer from a severe congenital encephalopathy.

**Methods:**

To identify the pathways mis-regulated by MeCP2 deficiency, microarray-based global gene expression studies were carried out in cerebellum of *Mecp2 *mutant mice. We compared transcript levels in mutant/wildtype male sibs of two different MeCP2-deficient mouse models at 2, 4 and 8 weeks of age. Increased transcript levels were evaluated by real-time quantitative RT-PCR. Chromatin immunoprecipitation assays were used to document *in vivo *MeCP2 binding to promoter regions of candidate target genes.

**Results:**

Of several hundred genes with altered expression levels in the mutants, twice as many were increased than decreased, and only 27 were differentially expressed at more than one time point. The number of misregulated genes was 30% lower in mice with the exon 3 deletion (*Mecp2*^tm1.1Jae^) than in mice with the larger deletion (*Mecp2*^tm1.1Bird^). Between the mutants, few genes overlapped at each time point. Real-time quantitative RT-PCR assays validated increased transcript levels for four genes: *Irak1*, interleukin-1 receptor-associated kinase 1; *Fxyd1*, phospholemman, associated with Na, K-ATPase;*Reln*, encoding an extracellular signaling molecule essential for neuronal lamination and synaptic plasticity; and *Gtl2/Meg3*, an imprinted maternally expressed non-translated RNA that serves as a host gene for C/D box snoRNAs and microRNAs. Chromatin immunoprecipitation assays documented *in vivo *MeCP2 binding to promoter regions of *Fxyd1, Reln*, and *Gtl2*.

**Conclusion:**

Transcriptional profiling of cerebellum failed to detect significant global changes in *Mecp2*-mutant mice. Increased transcript levels of *Irak1, Fxyd1, Reln*, and *Gtl2 *may contribute to the neuronal dysfunction in MeCP2-deficient mice and individuals with Rett syndrome. Our data provide testable hypotheses for future studies of the regulatory or signaling pathways that these genes act on.

## Background

DNA methylation is one of several strategies cells employ to regulate gene expression. In mammals, cytosine methylation at CpG dinucleotides is essential for tissue-specific gene expression, X-chromosome inactivation, genomic imprinting and silencing of transposable elements. The methylation signal is interpreted by a family of proteins that bind to methylated CpGs and recruit transcriptional repressor complexes. MeCP2, methyl-CpG-binding protein 2, was the first identified member of this family [1]. MeCP2 is an abundant nuclear protein that binds preferentially to heterochromatin regions on mouse chromosomes [1]. MeCP2 has a methyl-CpG-binding domain (MBD) that binds to methylated CpG dinucleotides adjacent to AT sequences [2]. Chromatin immunoprecipitation (ChIP) with anti-MeCP2 antibodies precipitated a large number of repetitive sequences as well as gene sequences [3,4]. *In vitro*, MeCP2 binds to promoters and represses transcription of diverse disease-related loci, including *FMR1, H19, CCNA1, BRCA1*, and several other genes [5-8]. Additional functions of MeCP2 may be unrelated to ^m^CpG binding: chromatin compaction, chromatin looping, alternative splicing, and nuclear reorganization [4,9-12].

*MECP2 *mutations are identified in ~ 90% of cases of classical Rett syndrome (RTT) [13-15]. RTT (OMIM 312750) is a neurological disorder characterized by apparently normal development for the first few months followed by developmental stagnation and regression, loss of purposeful hand movements and speech, truncal ataxia, stereotypic hand movements, deceleration of brain growth, autonomic dysfunction and seizures [16-19]. *MECP2 *is located at Xq28 and is subject to X inactivation. The classical RTT phenotype is manifest in heterozygous females that are mosaics for cells with either normal or mutant/absent MeCP2 protein. Males with a deleterious *MECP2 *mutation have no functional MeCP2 protein. Although apparently normal at birth, they display stagnant postnatal development, irregular respiration, central hypoxia and early death [20]. Currently, there is no effective therapy to prevent the developmental regression, autonomous nervous system dysfunction and loss of communication skills in RTT individuals. Gaining more information about MeCP2's functions and identifying the regulatory circuits that are disrupted by MeCP2 deficiency are essential steps for understanding the pathophysiology of RTT.

Originally, it was hypothesized that MeCP2 is a master regulatory protein that prevents inappropriate expression of a potentially large set of genes, and that in the absence of MeCP2, the expression of many genes would be altered. This hypothesis was not confirmed by microarray-based global expression studies. The numbers of mis-regulated genes were quite limited in different tissues from RTT individuals and mouse models with MeCP2 loss-of-function mutations. The lists of mis-regulated genes from different studies are essentially non-overlapping, and the search for specific MeCP2-target genes has turned up very few validated candidates [21-27].

We aimed to extend previous global gene expression studies in *Mecp2 *mutant mouse models by focusing on the cerebellum that was excluded in the previous work of Tudor et al. [23]. Gene expression patterns in the cerebellum are quite distinct from those in other brain regions in many mouse strains studied by Nadler et al. [28]. Expression of *Mecp2 *increases postnatally and is abundant in the adult cerebellum [29]. The truncal tremor observed in *Mecp2*-deficient mice [30,31] and the ataxia observed in RTT girls suggest cerebellar pathology. In mutant *Mecp2*-/Y mice, the cell bodies of cerebellar granule neurons are smaller and more densely packed [30]. Postmortem examination of the cerebella of five RTT females ranging in age from 7–30 years revealed loss of Purkinje cells, atrophy and gliosis [32]. These published data provided the rationale for our study design. To address the possibility of mutation-specific and developmental time-specific changes, we compared expression changes in males of two different *Mecp2*-mutant mouse strains and at three different postnatal time points.

Consistent with previous studies, we did not detect substantial up-regulation of a discrete set of genes. However, we observed moderately increased transcript levels mostly in 8 wk old mutant mice. For four genes, *Irak1, Fxyd1, Reln*, and *Gtl2*, increased transcript levels were validated by real-time quantitative RT-PCR. Attempts at replication of the data on independent samples and in different brain regions yielded variable results. Physical association of these genes with MeCP2 was confirmed by chromatin immunoprecipitation (ChIP) assays that documented MeCP2 binding to promoter regions of *Fxyd1, Reln*, and *Gtl2*. This study reports, for the first time, cerebellar expression profiles in *Mecp2*-mutants and proposes novel MeCP2 target genes that may contribute to the pathophysiology observed in RTT individuals and MeCP2-deficient mice.

## Methods

### Mouse breeding and sample collection

Females heterozygous for the J-allele (*Mecp2*^tm1.1Jae^) [30] were obtained from Rudolf Jaenisch and bred to BALB/cJ males. Females heterozygous for the B-allele (Mecp2^tm1.1Bird^) [31] were purchased from the Jackson Laboratory and were bred to C57BL/6J male mice. Tail DNA samples were genotyped for *Mecp2 *and *Sry *(for sex determination) as described [33]. *Sry *primers: F: TCCCAGCATGCAAAATACAG; R: TGGTCATGAAACTGCTGCTT. Only male mutant mice and their male wild-type siblings were used, because female heterozygotes are mosaic for MeCP2 deficiency and have variable expressivity of the mutant phenotype. For the microarray experiments and technical validation by qRT-PCR, we studied 23 mutants and 25 wild-type J-allele mice, and 32 mutant and 34 wild-type B-allele mice. For biological validation by qRT-PCR of a new set of samples, we used 20 mutant and 16 wild-type B-allele mice. Animals were euthanized with CO_2_. Cerebella and forebrains were snap frozen on dry ice. Total RNA was extracted using TRIzol Reagent (Invitrogen) and analyzed on a 1.5% agarose gel in MOPS buffer. RNA was concentrated and purified with YM30 microcon columns (Millipore). The Animal Care Committee of Stanford University approved all experimental procedures.

### Human brain samples

Brain samples from RTT individuals, including two hemizygous males with known MECP2 mutations, and non-RTT controls were obtained from the NICHD Brain and Tissue Bank for Developmental Disorders (UMB# 1238, 1420, 1748, 1815, 4516, and 4852) and from the Harvard Brain Tissue Resource Center (#b4160). We extracted total RNA from frozen frontal cortex using RNA Stat 60 (Tel-Test) or TRIzol reagent (Invitrogen). All human materials were obtained and studied under a protocol approved by the Stanford Human Research Protection Program.

### Expression cDNA microarrays

Microarrays were obtained from the Stanford Functional Genomics Facility (SFGF) [34]. These arrays contain 42,000 different transcripts that represent over 26,000 different genes. The transcripts vary in size and are PCR products from the RIKEN FANTHOM clone sets and a consortium of Stanford collaborators. The clone IDs on the cDNA microarrays are available from SFGF. Further information on the clones can be found at SOURCE [35].

### cDNA labelling and microarray hybridization

For preparation of reference RNA, we used a pool of 100 cerebella collected from wild-type C57BL/6J and BALB/cJ male mice ranging from P7 to P60 in age. Reference RNA (20 μg) was reverse transcribed using an anchored oligo(dT) primer and labeled with Cy3-dCTP using the CyScribe First-Strand cDNA Labeling Kit (Amersham Biosciences, Catalog # RPN6200). Total RNA (20 μg) from wild-type samples or mutant samples was reverse transcribed and labeled with Cy5-dCTP. Following purification with YM30 microcon tubes, labeled reference cDNA was combined with either labeled wild-type cDNA or labeled mutant cDNA. The combined reactions (reference and wild-type or reference and mutant sample) were applied to a cDNA microarray. Hybridizations were carried out at 65°C for 14–16 hr in a waterbath or hybridization oven. Blocking reagents were added according to the Brown Lab protocol [36].

### Microarray data analysis

Following hybridization, microarrays were washed and immediately scanned with a GenePix 4000B microarray scanner (Axon Instruments/Molecular Devices). Automatic and manual quality analysis of array spots was performed with Genepix Pro 4.0. All microarray data were deposited in the Stanford Microarray Database and normalized by total intensity. The Log (base2) of Red/Green Normalized Ratio (Mean) for each gene was retrieved and averaged only if the spot on the microarray was from the same microtiter plate (LUID – laboratory microtiter well ID). Genes were centered by the mean. Up to five replicate microarrays were evaluated for each mutant allele at each time point. There are 41,000 different spots on each microarray. Some of those spots are not "good" spots, meaning they do not pass the Genepix program filter and/or our manual evaluation. Spots do not pass our filter if the signal is too low, the spot does not have an even distribution of signal, or if there are any other experimental problems that deem the spot of poor quality. Some spots removed for low signal may imply that the gene is not expressed. Regardless of the reason, genes were excluded from our analysis if more than 85% of the spots representing a particular gene are filtered from our data set across replicate microarrays. After filtering, the number of genes that remained in our data set were: J-allele: 2 wk (25,365), 4 wk (30,657), 8 wk (29,774); B-allele: 2 wk (28,939), 4 wk (28,005), 8 wk (26,473). Differentially expressed genes were defined as genes whose expression is consistently (*P *< 0.05) altered across microarray experiments. Paired t-tests (Microsoft Excel) were performed by comparing the expression of each gene in a mutant sample to expression in its wild-type sibling. The data were analyzed with Microsoft Excel and SAM (Sequence Analysis of Microarrays) software [37]. Microarrays were centered and clustered using hierarchical clustering software available at the Stanford Microarray Database [38,39]. The cDNA microarray data can be accessed online via the SMD [88] or via the EBI ArrayExpress [89].

### Real-time quantitative RT-PCR (qRT-PCR)

Total RNA (2 μg) was treated with 2U DNase I (Ambion) and reverse transcribed with random hexamers as primers and Superscript II (Invitrogen). qRT-PCR was performed using an ABI 5700 (AME Bioscience) instrument. Melting curve analysis was performed for each reaction to ensure a single peak. Amplicons were visualized after electrophoresis on a 2% agarose gel to ensure presence of a single amplicon. Amplification of *β*-actin, *β *2-microglobulin, *Rps28 *and *RPS18 *transcripts served as RNA controls for relative quantitation (User Bulletin #2, Applied Biosystems). Mouse primers used (5' – 3'):

*Mecp2 *(F: TGACTTCACGGTAACTGGGAG; R: TTTCACCTGAACACCTTCTGATG),

*β-actin *(F: TGACCCTGAAGTACCCCATTGA; R: CCATGTCGTCCCAGTTGGTAAC)

*β 2m *(F: ACCCGCCTCACATTGAAATCC; R: CGATCCCAGTAGACGGTCTTG),

*Rps28 *(F: TAGGGTAACCAAAGTGCTGGGCAG; R: GACATTTCGGATGATAGAGCGG),

*Irak1 *(F: CAGAACCACCACAGATCATCATC; R: GGCTATCCAAGACCCCTTCTTC),

*Fxyd1 *(F: TCCATTCACCTACGATTACCACA; R: GAATTTGCATCGACATCTCTTGC),

*Reln *(F: CTGTGTCATACGCCACGAACA; R: GGGGAGGTACAGGATGTGGAT),

*Gtl2 *(F: GACCCACCTACTGACTGATGAACTG; R: GTGAAGACACAACAGCCTTTCTCC).

Human primers used (5' – 3'):

*FXYD1 *(F: AGGGACAATGGCGTCTCTTG; R: CGTAAGTGAACGGGTCGTGTT (NM_005031)

*RPS18 *(F: CTTTGCCATCACTGCCATTA; R: ACACGTTCCACCTCATCCTC)

### Chromatin Immunoprecipitation (ChIP)

We modified a protocol kindly provided by P. Farnham [40]. Brains of 4 wk-old male mice were isolated and minced to small pieces. Cross-linking reactions were performed at room temperature for 15 min in the presence of 1% formaldehyde in PBS. Nuclear extracts were prepared and chromatin was fragmented by sonication to produce DNA fragments of an average length between 500 bp and 1000 bp. After pre-cleaning with salmon sperm DNA/protein A agarose (Upstate), we incubated the nuclear extracts with anti-MeCP2 antibody (Upstate) at 4°C overnight. MACs Protein A Micro Beads (Miltenyi Biotec) were added to the reaction to allow the formation of magnetically labeled immune complexes. We purified the bound complexes using a microcolumn following the manufacturer's instructions. After reversal of cross-links and digesting with proteinase K, we isolated DNA fragments with a DNA purification kit (Qiagen). We performed 36 cycles of PCR with primers specific to the first ~ 500–1000 bp of the gene promoter region or to the DMR at the 5' region of the *Gtl2 *gene.

Primer pairs: *Fxyd1 *(F: GCATGTCCTGCTGTATGTCG; R: CAGCCAGGGTCAAGAAATGT), *Reln *(F: AAAGGGAGATTGGGTGACG; R: ACGTGCTTCTGGATGGTTTC), *Gtl2 *– A (F: TCCAACACGAAATTCTGCAA; R: TGCTGGTCAACATGAACCTC) amplifying nt 94579–94964 and the nested primers *Gtl2 *– B (F: CGGTCCACTAGGGCTTTTT; R: CCAGGTTTTTAGACCCCAGA) amplifying nt 94645–94775 of AJ320506.

## Results

### *Mecp*2-mutant mice differ in phenotype and expression of mutant *Mecp*2 allele

**Figure 1:**
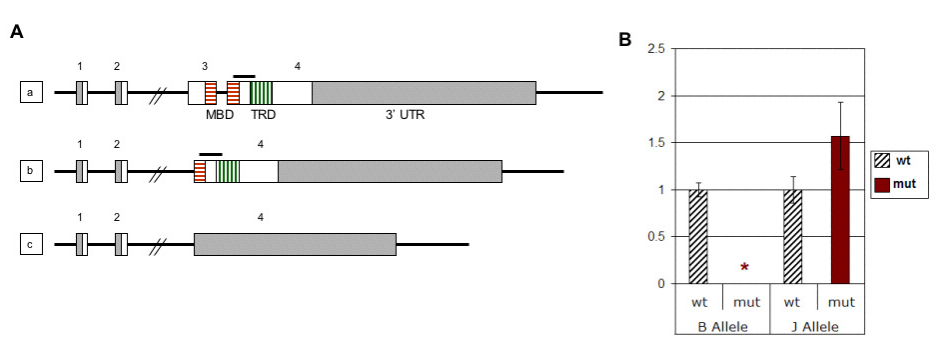
**Structure and expression of *Mecp2 *mutant alleles**. **A. *Genomic structure of *Mecp2 *mutations*. a. **Wild-type *Mecp2 *gene. MBD, methyl-CpG binding domain; TRD, transcription repression domain. White or hatched: coding sequence. Grey: non-coding parts of transcript. Exons 1 and 2 that are alternatively spliced, and intron 2, are not drawn to scale. Horizontal bar above exon 4 denotes location of the amplicon analyzed by qRT-PCR (in Figure 1B) **b. **The *Mecp2*^tm1.1Jae ^(J-allele) mutant has a deletion of exon 3 that encodes the N-terminal half of the MBD. **c. **In the *Mecp2*^tm1.1Bird ^(B-allele) mutant, exon 3, the coding portion of exon 4 and part of the 3'UTR are deleted. This larger deletion eliminates ~ 97% of the coding sequence. **B. *Real-time quantitative RT-PCR analysis of *Mecp2* expression in cerebellum*. **Using primers within the deleted region, *Mecp2 *transcript was undetectable in mutant mice with the B-allele, when four wild-type and four mutant samples were compared at 8 wk. This proves the specificity of the primers. In mice with the J-allele, mean *Mecp2 *expression was higher in the mutants, but this difference was not statistically significant (*P *= 0.11), when six wild-type and seven mutant samples were compared at 8 wk.

We examined gene expression in the cerebellum of two different *Mecp2*-mutant mouse models. The *Mecp2*^tm1.1Jae ^(J-allele) mutant has an in-frame deletion of exon 3 that codes for half of the MBD [30] (Figure 1A). Therefore, the expected mutant protein, if present, would be unable to bind methylated DNA. In contrast, the sequences encoding the transcriptional-repression domain (TRD), nuclear localization signals and C-terminal WW-binding domain, all encoded by exon 4, are retained. The second mutant, *Mecp2*^tm1.1Bird ^(B-allele), is deleted for exon 3 and the coding sequence of exon 4 [31]. In this allele, ~ 97% of the *Mecp2*-coding sequence is deleted, as well as a small part of the 3'UTR. No mutant mRNA was detected on Northern blot [31].

Asymptomatic females, heterozygous for a *Mecp2 *mutation and less than 6 months old, were bred to wild-type males. Offspring were genotyped for *Mecp2 *and *Sry *(for sexing). Cerebellar RNA was extracted from hemizygous males at mean ages of 2 wk, 4 wk and 8 wk. At 4 wk, 22% of mutant B-allele mice but none of the J-allele mice were symptomatic. Differences in body weight were observed in both sets of mice beginning at 4 wk, with the mutants being significantly smaller than wild-type littermates. At the "8 wk" time point, 80% of B-allele mice were symptomatic, while the range for J-allele mice extended from 10% at 7 wk to 71% at 9 wk. The most consistent abnormality observed in mutant mice was decreased mobility. At later stages, they developed tremors and hind-limb clasping and appeared poorly groomed.

Primers designed to amplify a region of exon 4 did not detect *Mecp2 *transcripts in B-allele mice as expected, but qRT-PCR studies revealed normal levels of the mutant *Mecp2 *transcript in 8 wk old mice with the J-allele (Figure 1B). Therefore, while the *Mecp2*^tm1.1Bird ^B-allele represents a true null mutation, the *Mecp2*^tm1.1Jae ^(J-allele) produces a stable mutant mRNA capable of directing synthesis of an internally deleted mutant protein that may have retained functions unrelated to ^m^CpG binding. By using whole brain extracts for Western blot analyses, we were unable to detect the predicted protein product of 309 amino acids made from the J-allele. All antibodies we tested cross-reacted with an ~ 55 kD unknown protein in all samples and, therefore, the putative J-allele product may have been obscured. Alternatively, it may have been degraded in the majority of neurons.

### Differentially expressed genes in *Mecp*2-mutant mice

**Figure 2:**
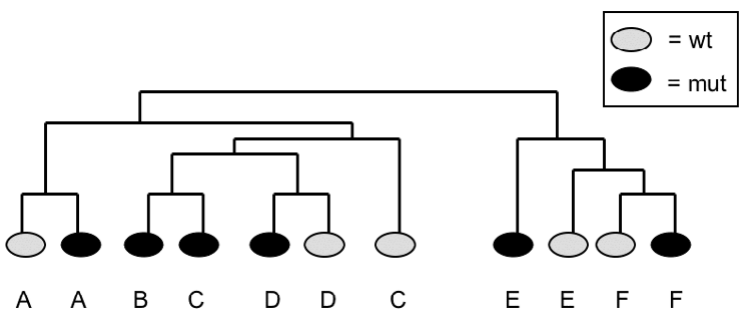
**Hierarchical clustering of microarray data from six mutant/wildtype sib pairs**. Male B-allele mutants at 8 wk of age, when most of them are symptomatic, tended to cluster with their wt sibs rather than with mutants of other litters. Litters are identified by A – F. The array of the litter B wild-type sib did not pass quality control standards.

**Figure 3:**
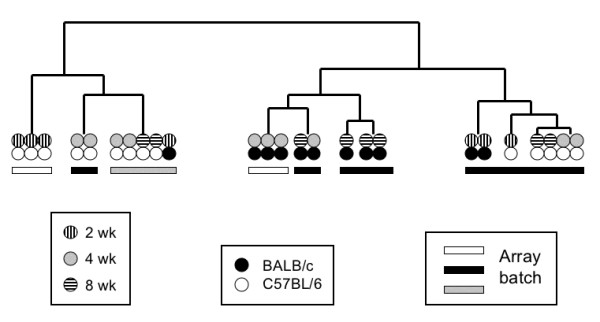
**Hierarchical cluster analysis of microarray data from wild-type sibs at three different ages**. Strain background had the greatest influence on clustering. BALB/c is the major background of the J-allele mice, while B-allele mice are on a C57BL/6 background. The effects of array batch, and to a lesser extent, age, are also apparent. We, therefore, compared only microarrays from littermates, at the same age and using the same array batch, in our search for differentially expressed genes.

To discover gene expression differences between mutant and wild-type cerebella, we performed microarray experiments of both mutant strains under the same conditions. Our microarrays contained 26,000 different cDNA clones spotted on glass slides (Stanford Functional Genomics Facility). Reverse-transcribed cDNA samples from either wild-type or mutant cerebella were hybridized to a cDNA microarray together with a reference sample consisting of pooled cerebellum cDNA. By carrying out hierarchical clustering of the microarray data, we found that several parameters greatly influenced the clustering. In comparing expression data from B-allele mice at 8 wk of age, littermates tended to cluster more than mutants of different litters (Figure 2). Clustering data from only the wild-type control mice illustrate the effects of age, strain background, and array batch (Figure 3). Therefore, in the final analysis, we only compared mutant expression arrays to wild-type arrays of mice that were from the same litter and only microarrays from the same batch. Pair-wise t-tests were performed to combine data from different wild-type/mutant pairs.

Consistent with previous expression microarray studies of whole brain or other brain regions, we found that loss of MeCP2 function does not lead to global mis-regulation of gene expression in cerebellum. Even without correction for multiple testing, the total number of differentially expressed genes with a p-value of < 0.05 was not significantly greater than would be expected by chance (Table 1). In B-allele mice, the number of genes with increased expression was slightly greater than would be expected by chance, but only at the 8 wk time point when more of the mutant mice used were symptomatic. The majority of changes reflect an increase of transcript levels, consistent with a relaxation of gene repression.

**Table 1:** Cerebellum gene expression microarray data

**Mutant**	**Age**	**Sib Pairs**	DEG*		**DEG Total**
		**(+/y, -/y)**	**up**	**down**	
J-allele	2 wk	5	292	2	294
	4 wk	3	126	105	231
	8 wk	5	441	399	840
					
B-allele	2 wk	3	380	262	642
	4 wk	4	423	45	468
	8 wk	3	615	234	849

*DEG: Differentially expressed genes with at least a 1.2 fold increase or decrease that are consistently changed for the given mutant and wild-type siblings (*P *< 0.05).

**Figure 4:**
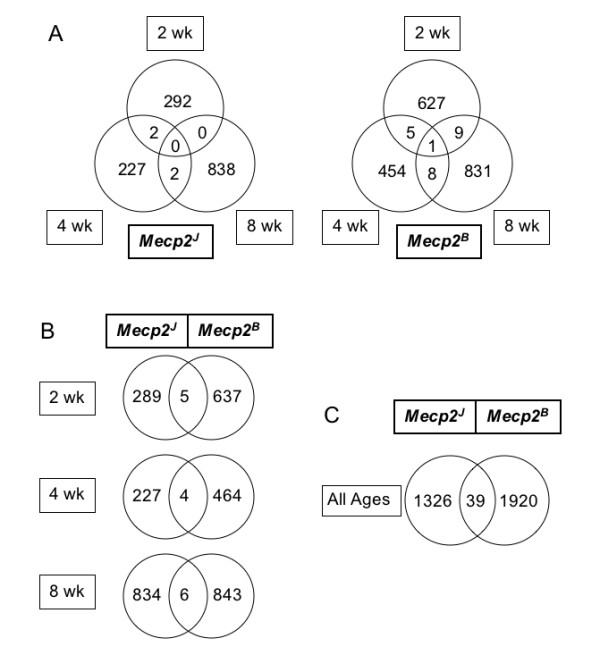
**Differentially expressed genes (DEG) in cerebellum microarray study**. **A. *Overlap of DEG at 2 wk, 4 wk, and 8 wk time points***. For both mutant alleles, genes whose transcripts were either increased (1.2-fold or higher) or decreased (0.8-fold or lower) at *P *< 0.05 are included. Transcripts in the overlap regions are described in Additional file 1. **B. *Overlap of DEG across both mutant alleles at three time points*. **The same criteria as in Figure 4A were applied. **C. *Overlap of DEG across all ages in both mutant strains***. The 39 transcripts include the 15 overlapping DEG from Figure 4B as well as genes that were significantly changed at one age in one mutant and at another time point in the other mutant. The list is provided in the Additional file 1.

To identify genes of potential biological significance, we studied the datasets from different time points and different alleles for common differentially expressed genes (DEG), but very few consistent differences were detected. The degree of overlap at 2 wk, 4 wk, and 8 wk for each mutant *Mecp2 *allele is very small (Figure 4A, Additional file 1). In male mice with the J-allele, no genes showed altered expression at all three time points. In B-allele mice, only one Riken clone (H3142G05) [35] was differentially expressed at all time points. Expression of this gene, later identified as *Tep1*, was increased on microarrays: 1.24, 1.52, and 1.25 fold at 2 wk, 4 wk and 8 wk, respectively. *Tep1*, which codes for telomerase-associated protein 1, was first discovered in *Tetrahymena *and is conserved from ciliates to humans. TEP1 is the RNA-binding protein of the telomerase ribonucleoprotein complex that also contains the catalytic reverse transcriptase subunit and an RNA component that serves as a template for the synthesis of telomeric repeats [41]. More recently, TEP1 was also identified as a protein component of vaults that are ribonucleoprotein complexes localized in the cytoplasm of cholinergic nerve terminals close to synaptic vesicles [42]. Specifically, TEP1 is required for the stable association of the vault RNA with the vault complex [43]. *Tep1 *expression was not significantly changed in mutant mice with the J-allele.

A total of 27 genes were differentially expressed at two different time points, four in the J-allele mutants and 23 in the B-allele mutants (Figure 4A). The gene displaying the greatest expression change is *Gtl2 (Gene-trap locus 2) *that was up-regulated 1.6 fold at 2 wk and 1.7 fold at 8 wk in B-allele mutants. Increased RNA levels were detected by two different clones on the cDNA microarrays at 2 wk (2210008A22) and 8 wk (1110014G20). The sequences are non-overlapping, but both represent the *Gtl2 *locus. *Gtl2 *is an imprinted gene producing a non-coding RNA of unknown function. The paternally expressed gene *Delta-like 1 *(*Dlk1*) and the maternally expressed *Gtl2 *gene are close to each other within an imprinted gene cluster on distal mouse chromosome 12 [44]. *Dlk1 *expression was not significantly changed in either mutant.

Since any expression changes that are relevant for the phenotype might be seen in both mutant strains, we determined the number of common expression changes *between *the two strains. In general, there is a low degree of overlap (39 out of 3285 genes) between DEGs in the B- and J-allele mutants, and only 15 genes overlap at the same time points (Figure 4B, Additional file 1). Potentially functionally relevant expression changes were observed in both mutants for several genes: *Smarcb1*, *Satb1*, *Fubp3 *and *Abt1. Smarcb1 *is a component of the SWI/SNF ATP-dependent chromatin-remodeling complex. Two studies have linked MeCP2 and the SWI/SNF chromatin-remodeling complex [5,45], although this connection is controversial [46]. SATB1 (special AT-rich sequence binding protein 1) is predominantly expressed in thymocytes and specifically binds to nuclear matrix attachment regions (MARs) [47]. It is involved in chromatin loop formation [48] and mediates formation of a higher-order transcriptionally active chromatin structure in activated T cells [49]. *Satb1 *is also expressed in spinal cord and distinct regions of the developing mouse brain in a mutually exclusive fashion with its close relative *Satb2 *[50]. Like SATB1, MeCP2 also binds to nuclear matrix-associated DNA repeat sequences in the human genome, and the chicken ortholog of MeCP2 is the MAR-binding protein ARBP [51]. In both mutant mouse models, increased expression was observed for two transcriptional activators, *Fubp3 *and *Abt1*, at 8 wk and for two other transcriptional regulators, *Rab8a *and *Prdm4*, at 8 wk and 2 wk, respectively (Additional file 1).

### Increased expression of *Irak1*, *Fxyd1*, *Reln*, and *Gtl2 *in *Mecp*2-mutant mice

**Figure 5:**
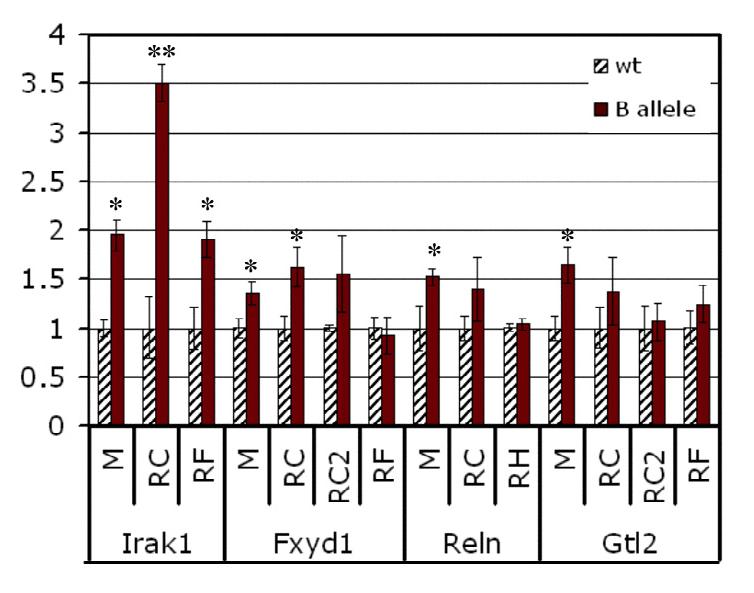
**Microarray and qRT-PCR results for select DEGs. ***Irak1, Fxyd1, Reln *and *Gtl2 *were selected for validation of expression changes by qRT-PCR analyses in B-allele mice. Cerebellum microarray expression data (M) are compared with qRT-PCR data from cerebellum (RC), forebrain (RF) and hippocampus (RH). For RC of *Irak1, Fxyd1 *and *Gtl2*, the 8 wk samples from the microarray analysis were used. RC2 data are derived from an independent set of litters at 7.5 to 8 wk. Error bars represent SEM. One asterisk indicates *P *< 0.05, two asterisks indicate *P *< 0.001. *Irak1 *expression was markedly increased in the cerebellum of 8 wk B-allele mice on microarray (2-fold, *P *= 0.011, four mutant/wild-type pairs) and qRT-PCR (3.5 fold, *P *< 0.001, four mutant/wild-type pairs) as well as in the forebrain of a different set of mice at 7.5 wk (1.9 fold, *P *< 0.05, three mutant/five wild-type). *Fxyd1 *expression was elevated 1.4-fold on microarrays (*P *< 0.05, five mutant/wild-type sibs) and qRT-PCR (RC) **(***P *= 0.053, four mutant/wild-type sibs). The RC2 data (five mutant/three wild-type) were more variable and yielded *P *= 0.16. *Reln *displayed an ~ 1.5 fold increase in expression on microarrays (*P *= 0.03, four mutant/wild-type pairs) and qRT-PCR (*P *= 0.06, five mutant/wild-type pairs) at 2 wk (RC) and was not changed in hippocampus (RH). On microarray, *Gtl2 *expression was increased at 8 wk, but by qRT-PCR we found highly variable *Gtl2 *expression in cerebellum (RC and RC2). *Gtl2 *expression was not significantly increased (*P *= 0.29, eight mutant/eight wild-type) in the forebrain.

For further studies, we selected four genes based on a fold change of at least 1.4 and consistency in the directional change over the majority of microarrays: *Irak1*, *Fxyd1, Reln, and Gtl2 *(Table 2). Expression of *Irak1*, encoding interleukin-1 receptor-associated kinase 1, was increased in all mutant/wild-type comparisons at 8 wk in B-allele mice. In real-time qRT-PCR experiments, *Irak1 *expression was increased 3.5-fold in the mutant relative to the wild-type 8 wk B-allele cerebellum samples and 1.9-fold in the forebrain (RC and RF, Figure 5). In the microarray data, *Irak1 *expression was not significantly altered at other ages, nor was it altered in J-allele mice. The *Irak1 *gene is located only 3 kb distal to *Mecp2*. Therefore, this expression change may reflect the altered genomic environment. For example, an element that negatively influences *Irak1 *expression could be deleted in the B-allele, but not in the J-allele that has a much smaller deletion.

**Table 2:** Genes with increased expression levels on microarrays in B-allele mutants, supported by qRT-PCR and/or ChIP Data

**Gene Symbol**	**Gene Name**	**Function**	**Genomic Locus**	**Age**	**Fold Increase**
*Irak1*	Interleukin-1 receptor-associated kinase 1	Proinflammatory cytokine response, neuroprotection	X A7.3	8 wk	2.2
*Fxyd1*	FXYD domain-containing ion transport regulator 1	Ion transport regulator for Na, K-ATPase	7 B1	8 wk	1.4
*Reln*	Reelin	Neuronal layer formation, cell-cell interactions	5 A3-B1	2 wk	1.5
*Gtl2*	GTL2, imprinted maternally expressed untranslated RNA	Growth suppressor, Host gene for snoRNAs and microRNAs	12 F1	2 wk 8 wk	1.6 1.7

*Fxyd1 *encodes the FXYD domain containing ion transport regulator 1, also called phospholemman. *Fxyd1 *expression was consistently increased in all microarray experiments (average 1.4 fold) of B-allele mice at 8 wk (Table 2). The results were confirmed by qRT-PCR analysis on the same cerebellum samples (RC, Figure 5). Biological replication by qRT-PCR on a different set of B-allele mice (RC2; 3 wt, 5 mut) yielded more variable results between litters resulting in large SEM error bars and a *p*-value of 0.13. *Fxyd1 *expression in forebain samples from six different litters showed no difference between mutant and wild-type samples, but *Fxyd1 *expression is normally very low in forebrain.

*Reln*, encoding the extracellular signaling molecule reelin, is highly expressed in the developing cerebellum. Reelin is essential for proper neuronal lamination and synaptic plasticity. We found increased *Reln *transcript levels (1.5 fold) in four microarray experiments of B-allele samples at 2 wk (M, Figure 5). *Reln *expression was also increased in qRT-PCR experiments (RC), but this increase is not statistically significant as there was substantial variation among the samples tested (Figure 3). No difference was seen in hippocampus when 5 wild-type and 7 mutant samples were compared (RH, Figure 5).

*Gtl2*, encoding an imprinted maternally expressed non-translated RNA, is the mouse homolog of *MEG3*, maternally expressed gene 3, in humans. A study evaluating the transcripts encoded by *Gtl2 *identified 13 different splice variants [52]. Our microarray data revealed significantly increased *Gtl2 *expression at 2 wk and 8 wk in B-allele mice (M, Figure 5). By qRT-PCR assays, the expression increase was present, but not significant in the same samples (RC) and in forebrain (RF, Figure 5). Cerebellum studies of a new set of mice (3 wt, 5 mut), however, showed no difference (RC2, Figure 5).

### MeCP2 binds to the promoters of *Fxyd1 *and *Reln *and the promoter and differentially-methylated region of *Gtl2 in vivo*

To determine if MeCP2 binds directly to the potential target genes, we performed chromatin immunoprecipitation (ChIP) assays on brain tissues. ChIP involves cross-linking genomic DNA to proteins that are bound to it. Subsequently, DNA-protein complexes are isolated by immunoprecipitation with a MeCP2 antibody and cross-links are reversed. The MeCP2 bound sequences are used as templates for semi-quantitative PCR to determine whether the gene region of interest is binding to MeCP2 [40].

**Figure 6:**
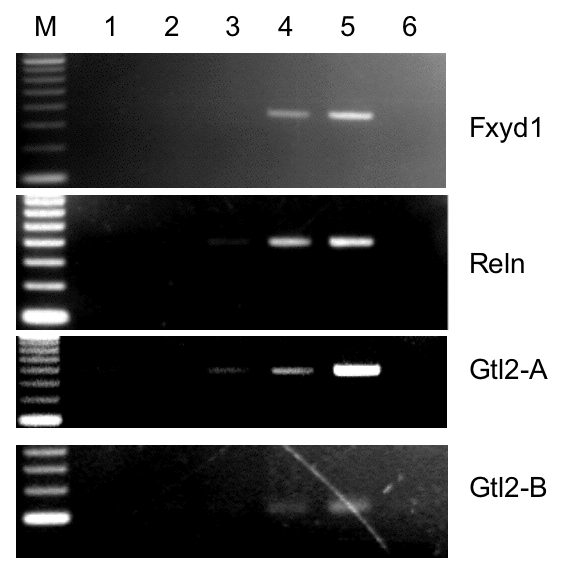
**Chromatin immunoprecipitation (ChIP) analysis of differentially expressed genes**. Whole brain nuclear extracts from 4 wk old wild-type and B-allele mutants were used for ChIP. Lane M, 100 bp ladder; lane 1, *Mecp2*-mutant chromatin precipitated with anti-MeCP2 (negative control); lane 2, wild-type chromatin precipitated with no antibody present; lane 3, wild-type chromatin precipitated with rabbit IgG, lane 4: wild-type chromatin precipitated with anti-MeCP2, lane 5: input DNA, lane 6: no DNA control. The precipitated DNA was amplified with primers specific for the promoter regions of *Fxyd1 *and *Reln*, and for the promoter/DMR of *Gtl2*. The Gtl2-B amplicon is located within the Gtl2-A amplicon. Precipitation with anti-MeCP2 shows enrichment relative to the IgG and no antibody controls, indicating that MeCP2 is associated with the designated regions *in vivo*. As negative controls, PCR was performed using primers covering regions that do not interact with MeCP2 for each ChIP reaction (not shown).

When we used primers specific for the 500–1000 bp *Fxyd1 *or *Reln *promoter regions, ChIP results indicated specific MeCP2 interaction in normal brain extracts (Figure 6). Amplification was enriched only in the presence of MeCP2 antibody (Figure 6, lanes 4). There was no, or much less, amplification of the DNA fragments when ChIP was performed either with nuclear extract from *Mecp2*-mutant mouse brain (lanes 1), or without MeCP2 specific antibody (lanes 2), or with non-specific antibody (IgG) (lanes 3). The results suggest that MeCP2 is part of a complex that is located at the promoter regions of these genes in normal brain and that this complex may not form in brain of *Mecp2*^tm1.1Bird^ mutant mice.

*Gtl2 *is an imprinted transcript whose maternal-specific expression is controlled by a differentially-methylated region (DMR) that overlaps the promoter region. MeCP2 binding was systematically analyzed using overlapping primer sets, each of which covers 250–400 bp of a region that contains the *Gtl2*-DMR (AJ320506, nt 92340–95350). MeCP2 binding activity was detected upstream of exon 1 and extending through exon 1 into intron 1 (AJ320506, 93720–95350) (Figure 6). This 1.6 kb region contains 63 CpG dinucleotides, many of which are differentially methylated.

### *FXYD1 *expression in human brain with MeCP2 deficiency

**Figure 7:**
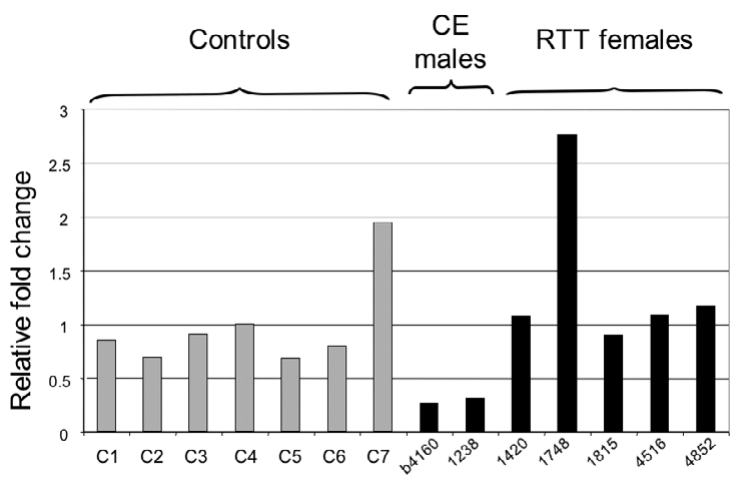
**Relative *FXYD1 *expression in human frontal cortex**. Quantitative real-time RT-PCR analyses were carried out on frozen frontal cortex samples from seven unaffected controls (C1 – C7), two *MECP2*-mutant males with congenital encephalopathy (CE) and five females diagnosed with RTT, with or without identified *MECP2 *mutations. The mutant samples are identified by their brain bank numbers. *FXYD1 *expression was normalized to *RPS18*, a constitutive ribosomal protein gene that is expressed at a similar level in brain. To calculate relative fold changes, control sample C4 was arbitrarily set at 1. All samples were run in triplicate on the same plate.

Recently, Deng et al (2007) [53] identified *FXYD1 *as a target of MeCP2 by increased expression in the frontal cortex of RTT individuals and of *Mecp2*-mutant mice, with no changes observed in the cerebellum. While these authors confirmed by ChIP that MeCP2 binds to the *FXYD1 *promoter, their expression results were the opposite of our findings in mouse brain. Therefore, we examined *FXYD1 *expression in human brain samples from five RTT females, two males with congenital encephalopathy (CE) and documented *MECP2 *mutations, and seven non-RTT controls (Figure 7). We used real-time quantitative RT-PCR with the same primers as Deng et al. [53]. We normalized *FXYD1 *expression levels to *RPS18 *expression rather than to rRNA expression. The ages of the donors differed only slightly, with controls in their 30 s and RTT females in their 20 s. The two CE males were between 1 and 2 years old. No *MECP2 *mutation had been identified in the single female RTT sample (UMB# 1748) with an increased *FXYD1 *transcript, but a brain contusion lesion was seen on autopsy. Our results do not confirm an increase in *FXYD1 *expression in frontal cortex of MeCP2-deficient humans, and they agree with our data on *Mecp2*-mutant mice. If MeCP2 were responsible for silencing *FXYD1 *in the forebrain, one would expect to observe a major increase in hemizygous males with CE who have no MeCP2 activity at all, as compared to RTT females who are mosaic for MeCP2-deficient and normal cells.

## Discussion

The aim to discover abnormally expressed genes in MeCP2-deficient mice is two-fold: to reveal direct targets of MeCP2 modulation and to identify components of neuronal pathways and circuits that are deregulated in affected brain regions. The ultimate goals are to understand the pathophysiologic processes that lead to morbidity in RTT and to design rational therapies to prevent or revert the functional abnormalities. To reduce microarray data noise caused by cellular heterogeneity of tissues, we focused on the cerebellum, a part of the brain not previously studied. We compared expression profiles in two different MeCP2-deficient mouse strains that differ on the molecular level, the *Mecp2*^tm1.1Bird ^(B-allele) and *Mecp2*^tm1.1Jae ^(J-allele). Furthermore, we compared expression profiles at three postnatal ages that span different developmental stages in the cerebellum. At two weeks, granule cells are still mitotic and migrating to their final position in the internal granule layer. At four and eight weeks, granule cells are post-mitotic and have begun forming synapses with neurons in the cerebellum and other brain regions.

Consistent with previous studies on forebrain and hippocampus samples by Tudor and colleagues [23], we did not observe major expression changes of a large number of genes in the cerebellum. MeCP2-deficiency does not significantly alter the cerebellar transcriptome on a global scale. This is remarkable given that the vast majority of cells in the cerebellum are granule cells that had shown morphological abnormalities in J-allele mice [30]. Any significant overexpression of genes specifically in granule cells should have been detectable, as it would not be averaged out by cell type heterogeneity. Our results should caution investigators who engage in cell-type specific profiling studies. Also, no large (> 3 fold) changes were detected for any single gene. The changes in cerebellum transcript levels tended to be small (1.5 – 2 fold), but increases greatly outnumbered decreases. Overall, a number of genes with increased transcript levels greater than expected by chance were observed only in B-allele mice at 8 wk. There are several possible explanations for these findings.

First, the mutations are different. While B-allele mice are null at the *Mecp2 *locus, the J-allele mutation consists of an in-frame deletion of exon 3 that produces a stable mutant transcript. The putative mutant protein, which lacks the MBD domain, but has an intact nuclear localization signal, transcription repression domain and C-terminal region, may have maintained certain functions. Some evidence has been reported for a truncated MeCP2 protein in the J allele mice. Luikenhuis et al. [54], saw diffuse nuclear staining with a MeCP2 antibody and speculated that this probably arose from the detection of the truncated protein. This interpretation is in line with the original report of the J-allele mice [30], where Western blot showed a smaller band that was interpreted to represent the truncated protein, even though the wild-type samples had a protein of apparently the same size, but at a lower level of intensity. The possibility cannot be excluded, however, that the diffuse nuclear staining was caused by a cross-reacting protein that does not localize to DAPI-positive foci. Using tissue microarrays to study the mosaic composition of *Mecp2*-expressing and non-expressing cells in female mice, Braunschweig et al. [55], reported a background immunoreactivity with a C-terminal antibody in male J-allele mice that was apparently not present in mice with the B-allele. These results would argue in favor of the presence of an internally deleted mutant protein present in the J-allele brain, although the intensity was rather low.

Second, the mice were on different strain backgrounds: B-allele mice on C57BL/6 and J-allele mice on a mixed background, mostly BALB/cJ. Our hierarchical clustering data clearly demonstrated a strong effect of strain background (Figure 3). Random mixed strain background noise should be overcome, to some extent, by our comparing only wild-type and mutant sib-pairs and by including a sufficiently large number of sib-pairs (16 for the B-allele and 13 for the J allele).

Third, 80% of the B-allele mice at 8 wk were symptomatic, while fewer of the J-allele mice were showing symptoms. When the disease process in the brain is further advanced, the expression changes may be caused by pathologic events far downstream of direct MeCP2 action.

Although the global level of gene expression is not significantly altered, that does not preclude the possibility that some of the genes may have changed expression levels that are biologically relevant. Therefore, we focused on whether differentially expressed genes showed a similar change in other brain regions and in a different set of mice (biological validation), which is a more relevant evaluation than "statistical" validation. We chose four genes, *Irak1*, *Fxyd1*, *Reln*, and *Gtl2*, whose expression levels were increased in the most consistent fashion. By ChIP analyses, we show that MeCP2 binds to the promoters of *Fxyd1 *and *Reln *and to a differentially methylated region of the imprinted gene *Gtl2 *in normal mouse brain, suggesting that these genes may be direct targets of MeCP2.

### Irak1

The interleukin-1 receptor-associated kinase 1 gene revealed the largest significant expression increase of any gene, in cerebellum (2.0-fold on microarray and 3.5-fold on qRT-PCR assays) and in forebrain (1.9-fold), but only in B-allele mice. *Irak1 *is located ~ 3 kb downstream of *Mecp2 *in the same transcriptional orientation (UCSC Genome browser, Feb 2006 assembly). This suggests that a negative regulator of *Irak1 *could have been lost with the B-allele deletion, or that the altered local chromatin conformation leads to increased *Irak1 *expression. The less likely possibility that MeCP2 directly regulates the expression of *Irak1 *would assume that the J-allele has maintained a negative regulatory function, since *Irak1 *expression is not increased in J-allele mutants. This allele-specific difference raises the concern that the increased *Irak1* expression may be responsible for some dysregulated genes in the B-allele mice that are not dysregulated in the J-allele mice. To identify *bona fide *MeCP2 targets we, therefore, focused on genes whose promoters bind MeCP2 in ChIP assays. Nevertheless, any studies done with the B-allele mice, at any level, from behavior to histology, should be considered in the light of possible downstream effects of *Irak1 *overexpression.

IRAK1 belongs to the ser/thr protein kinase family, *pelle *subfamily. The protein is comprised of a death domain in the N-terminus, a central serine/threonine kinase domain, and a C-terminal serine/threonine rich region. Once phosphorylated, IRAK1 recruits the adapter protein PELI1 and causes activation of Toll-like-receptor-mediated intracellular signaling pathways. Three different splice variants in humans and mice differ in their relative abundance in brain, and differential splicing of IRAK1 may correlate with the aging process [56]. Our qRT-PCR assay amplified all three splice forms. Given the postulated role of MeCP2 in alternative splicing [11], it might be of interest to evaluate the relative abundance of the different *Irak1 *splice forms in *Mecp2*-mutant B-allele brain.

### Fxyd1

In wild-type mice, *Fxyd1 *expression increases drastically from 2 wk to 8 wk in the cerebellum (our microarray data). In *Mecp2 *mutant mice, microarray and qRT-PCR studies revealed a significant increase of *Fxyd1 *transcripts at the 8 wk time point in B-allele mutants when compared to wild-type littermates. ChIP analyses confirmed the binding of MeCP2 to the *Fxyd1 *promoter. We, thus, have identified *Fxyd1 *as a validated target of MeCP2 binding. In contrast to a recent report by Deng et al. [53], however, *Fxyd1 *transcripts were not increased in the mutant forebrain where expression levels are normally very low. Attempting to replicate the reported studies in humans, we have used qRT-PCR to measure *FYXD1 *expression in frontal cortex from male and female individuals with *MECP2 *mutations and unaffected controls. We found no evidence for increased expression in the mutant samples. Therefore, our data in humans and mice do not support the hypothesis that MeCP2 is responsible for keeping *FYXD1*/*Fxyd1 *expression low in the majority of cells of forebrain. A small increase of expression in a neuronal subtype could have gone undetected, but the large increases reported by Deng et al. [53] in RTT frontal cortex are not confirmed by our results.

The protein product, FXYD1, also called phospholemman (PLM), is a small molecule with a single transmembrane domain and a member of the FXYD family of small ion transport regulators. FXYD proteins regulate Na-K-ATPase activity in a tissue- and isoform-specific way. Each FXYD gene is expressed in one or more specific tissues [57]. FXYD1 is a major membrane phosphoprotein in heart and muscle. In brain, FXYD1 is predominantly found in the cerebellum, specifically in the molecular layer, in Purkinje neurons, and in axons traversing the granule cell layer, as well as in the choroid plexus [58]. The protein forms a helical structure and inserts into lipid membranes in a *trans*-bilayer fashion [59]. FXYD1 tetramers form ion channels selective for K+, Cl-, and taurine that are physically associated with the Na-K-ATPase [60]. Recent studies revealed that FXYD1 also inhibits the cardiac Na+/Ca2+ exchanger (NCX1) [61]. Overexpression of FXYD1 in adult cardiac myocytes acutely alters contractility as a function of extracellular Ca+ concentration, and *Fxyd1 *knock-out mice have cardiac hypertrophy [62]. Alteration of *Fxyd1 *expression in the heart of *Mecp2*-deficient mice and humans could conceivably contribute to cardiac dysfunction and sudden death.

Another member of the FXYD family, FXYD4/CHIF (corticosteroid hormone induced factor) was initially isolated as a glucocorticoid response gene [63]. Interestingly, two putative MeCP2 target genes, *Sgk1 *and *Fkbp5 *are also glucocorticoid-inducible and were found to be upregulated in 8 wk old B-allele mice studied previously [25]. Furthermore, in the *Mecp2*^308 ^mouse model of MeCP2 deficiency, circulating corticosteroid levels were increased in response to stress, and the gene for corticotropin-releasing hormone (*Crh*) was overexpressed in brain regions, leading to the conclusion that the mutant mice have an enhanced corticosteroid response to stress via increased CRH signaling [64]. Future studies should evaluate whether *FXYD1*, like *FXYD4*, is also corticosteroid inducible.

### Reln

Our identification of *Reln *as a primary target of MeCP2 is supported by the following observations: *Reln *transcript levels were increased in microarray and qRT-PCR experiments comparing cerebella from a total of 12 wild-type and 9 mutant mice. And the expression change was seen at the earliest time point (2 wk) when all mice were asymptomatic. No expression change was found in hippocampus. ChIP assay on whole brain documented that MeCP2 binds to the *Reln *promoter in normal mice. The proximal promoter of *Reln *is highly CpG-rich and methylated in whole brain extracts [65]. Multiple hypermethylation sites at the *Reln *promoter can be induced by injection of L-methionine. And in a methionine-induced model of schizophrenia, MeCP2 was shown to bind to the *Reln *promoter [66,67].

Reelin is a large extracellular protein that is produced by discrete populations of cells in the brain. It acts through the extracellular milieu on neighboring target cells. Until recently, the primary function of reelin was thought to involve regulation of neuronal migration during fetal brain development, by reelin providing an architectonic signal for the guidance of migrating neurons [68,69]. Reelin-expressing cells include the Cajal-Retzius (CR) cells in the cortical marginal zone, that are the first neurons to express *Mecp2 *in development, and cerebellar granule cells. MeCP2-deficient mice have a thinner cortex and more densely packed neurons that could result from migratory defects.

*Reln *expression continues in the adult, in particular in a subset of GABAergic neurons of the forebrain, as well as in the olfactory bulb [70]. Reelin can modulate synaptic plasticity and enhance long-term potentiation (LTP) in adult hippocampal cultures [71]. The signaling pathway that reelin acts on involves two high-affinity binding receptors, the very low density lipoprotein receptor (VLDLR) and the apolipoprotein E receptor 2 (ApoER2) [72]. Binding of reelin to VLDLR and ApoER2 triggers phosphorylation of the cytoplasmic adaptor protein disabled 1 (Dab1) by *Src *family kinases, in particular *Fyn*. This series of reactions is required for cortical layer formation and for hippocampal dendrite development [73]. In addition, cyclin-dependent kinase 5-dependent signals are required for the function of reelin not only in neuronal migration but also in synaptic transmission [74]. The reelin signaling pathway is also involved in modulating learning and behavior [75]. Reelin can regulate NMDA-type glutamate receptor activity and potentiate calcium influx through NMDA receptors in neuronal cultures [75]. Sinagra et al. [76] documented a reelin-controlled change in subunit composition of NMDA glutamate receptors during maturation. Reelin signaling through ApoE receptors plays an essential role in synaptic plasticity and function in the adult brain [77].

Interestingly, BDNF, brain-derived neurotrophic factor, a primary target of MeCP2 [78,79] regulates reelin production in cortical neurons. *Bdnf *-/- mice have elevated reelin levels in Cajal-Retzius cells. Overexpression of BDNF produces brain abnormalities similar to the phenotype of *reeler (rl) *mutant mice. This phenotype is preserved in slice cultures of hippocampus [80]. Furthermore, in *in vitro *co-culture systems, exogenous reelin caused dispersal of chain-migrating interneuron precursors in the olfactory bulb, suggesting that overexpression of reelin may affect neuronal migration [81].

We hypothesize that *Reln *overexpression is responsible for part of the phenotype of MeCP2-deficient mice, and that this phenotype is caused by abnormal neuronal migration in the developing brain of MeCP2-deficient mice and by dysregulation of reelin signaling pathways, thus disturbing synaptic function, in postnatal brain. To test this hypothesis, we have initiated genetic interaction studies. We aim to reduce the level of *Reln *expression in the *Mecp2 *mutant background by crossing *rl*+/- heterozygous males to *Mecp2*+/- female mice. We have preliminary evidence that the onset of morbid phenotypes is delayed and lifespan prolonged in some of the *Mecp2*Y/- ;*Rln*+/- double-mutant mice, when compared to their *Mecp2*Y/- ;*Rln*+/+ littermates.

### Gtl2 (gene trap locus 2)

By microarray analysis, we identified *Gtl2*, called *MEG3 *(Maternally-expressed gene 3) in humans, as a significantly increased transcript in mutant cerebellum samples. qRT-PCR confirmed increased expression, although not significant due to large biological variation in different litters, in cerebellum and forebrain. *Gtl2 *encodes a maternally expressed imprinted non-translated RNA of unknown function. *Gtl2 *is expressed in most tissues, but most highly in brain. It lies within a cluster of imprinted genes on chromosome 12 that is conserved on distal 14q in human. Alternatively-spliced *Gtl2 *transcripts extend to include intron-encoded C/D box snoRNAs [82] and microRNAs [83,84]. Thus, *Gtl2 *might function as a host gene for these small RNAs. In our qRT-PCR assays, the increase in *Gtl2 *expression in cerebellum was less striking, but *Gtl2 *expression was consistently increased in the forebrain of *Mecp2*-deficient mice.

Two previous studies have identified *Gtl2 *as a possible target of MeCP2. An expression microarray study on whole brain of one B-allele mouse revealed a 1.9-fold increase in *Gtl2 *expression [25]. Independently, by using a variant of differential display technology and qRT-PCR confirmation, Kriaucionis and colleagues [27] detected increased *Gtl2 *expression in whole brains of B-allele mice. Since the increase was significant only at late symptomatic stages, the authors suggested that it may represent a secondary consequence of the disease pathology. In contrast, our data show a similar increase in *Gtl2 *expression at 2 wk and at 8 wk, albeit detected by two different cDNA clones on the array. *Gtl2 *has a CpG-rich promoter region that is unmethylated on the maternal allele and becomes hypermethylated on the paternal allele after fertilization [85,86]. By ChIP assay, we found that MeCP2 binds *in vivo *to this region with binding sites extending from 5' of exon 1 into intron 1, raising the possibility that it may play a role in silencing of the paternal allele. Alternatively, MeCP2 may bind to methylated CpG sites on the active maternal chromosome and thus regulate *Gtl2 *repression in certain brain regions or cell types. Future studies of allele-specific expression levels will address these possibilities.

Considering other previously reported candidate MeCP2 target genes that are represented on our microarrays: Only *Snrp70*, reported to be increased by qRT-PCR in whole brain from late symptomatic B-allele mice (1.44 fold, p = 0.04)[27], showed increased expression (1.5-fold) in cerebellum of 8 wk B-allele mice in our study. None of the following genes had significant expression changes in cerebellum: *Uqcrc1 *[27], *Bdnf *[87], *Dlx5 *[4], *Fkbp5 *and *Sgk1 *[25]. This is not surprising given that some of these genes may be primarily expressed in other brain regions and/or only in certain neuronal sub-types. Or transcript levels may be variable, when expression is modulated by activity state as for *Bdnf *[78,79]. Our experience with biological validation and hierarchical clustering of independent samples taught us about the mitigating effects of different sibship identities. We need to consider that the different mothers are *Mecp2*+/- heterozygotes, having the genotype of females with RTT. Although not obviously symptomatic yet, their variable X-inactivation status could influence expression profiles in their offspring.

## Conclusion

Cerebellar gene expression profiling of two *Mecp2*-mutant mouse models at three postnatal time points revealed no significant global changes. *Irak1*, located close to *Mecp2*, showed the most significant expression increase, but this may be specific to the large deletion in B-allele mice. We identified three novel direct MeCP2 target genes by increased expression and binding of MeCP2 to their promoters. Variable results in different litters do no preclude the potential importance of these genes. To evaluate the contributions of these mis-regulated genes to the synaptic dysfunction and neuropathologic phenotype observed in MeCP2-deficient humans and mice, further studies using higher cytological resolution, laser-capture dissection and immunohistochemical assays, as well as genetic interaction studies and functional assays of the affected pathways, will be needed.

## List of Abbreviations

RTT, Rett syndrome; MeCP2, methyl-CpG binding protein 2; DEG, differentially expressed genes; ChIP, chromatin immunoprecipitation

## Competing interests

The author(s) declare that they have no competing interests.

## Authors' contributions

CJ carried out the microrray and qRT-PCR studies, and the statistical analyses, and drafted the manuscript. HHL carried out the chromatin immunoprecipitation assays, validation studies and qRT-PCR of human brain samples. HCK participated in mouse breeding, genotyping and experimental design. UF conceived of the study, participated in its design, interpreted the results and prepared the final manuscript. All authors read and approved the final manuscript.

## Pre-publication history

The pre-publication history for this paper can be accessed here:



## Supplementary Material

Additional File 1 Differentially expressed genes (DEG) illustrated in Figure 4. This table lists the DEGs in the overlaps shown in Figure 4, their fold changes in B-allele and J-allele mice at different ages and their Clone IDs. Click here for file
