# Supplementary material for: Cerebellar gene expression profiles of mouse models for Rett syndrome reveal novel MeCP2 targets
Source: BMC Med Genet. 2007 Jun 20;8:36. doi: 10.1186/1471-2350-8-36 (PMC1931432; doi:10.1186/1471-2350-8-36)
Supplement: Additional File 1 — Differentially expressed genes (DEG) illustrated in Figure 4. This table lists the DEGs in the overlaps shown in Figure 4, their fold changes in B-allele and J-allele mice at different ages and their Clone IDs. [file 1471-2350-8-36-S1.doc]

**Additional file 1.** Differentially expressed genes (DEG) illustrated in Figure 4

| **DEG in B-allele mice at 2 wk (2B) and 4 wk (4B)** | | | | | |
| --- | --- | --- | --- | --- | --- |
| **Gene Symbol** | **Gene Name** | **Function** | **2B Fold Change** | **4B Fold Change** | **Clone ID** |
| *Jak3* | Janus kinase 3 | tyrosine kinase | 1.42 | 1.21 | IMAGE:457114 |
| *Flnb* | Filamin, beta | actin binding, myogenesis | 1.59 | 1.24 | UI-M-AO0-abz-e-02-0-UI |
| *Tep1* | Telomerase associated protein 1 | telomerase protein component | 1.24 | 1.52 | H3142G05 |
| *Heca* | Headcase homolog (Drosophila) | unknown | 1.24 | 1.27 | UI-M-AO0-acf-h-07-0-UI |
| NA | BG065979 | unknown | 1.30 | 1.30 | H3037E08 |
| NA | BG069270 | unknown | 1.40 | 1.71 | H3074E11 |
| **DEG in B-allele mice at 2 wk (2B) and 8 wk (8B)** | | | | | |
| **Gene Symbol** | **Gene Name** | **Function** | **2B Fold Change** | **8B Fold Change** | **Clone ID** |
| *Gtl2* | Gene trap locus 2 | imprinted ncRNA | 1.60 | 1.70 | 2210008A22  1110014G20 |
| *Tep1* | Telomerase associated protein 1 | telmerase protein component | 1.24 | 1.25 | H3142G05 |
| *Cdk4* | Cyclin-dependent kinase 4 | Cell cycle | 1.33 | 1.21 | 2700002H18 |
| *App* | Amyloid beta (A4) precursor protein | beta galatosidase | 1.33 | 1.46 | H3124B09 |
| *Mical2* | Microtubule associated monoxygenase, calponin and LIM domain containing 2 | cytoskeletal network | 1.34 | 1.29 | H3021B09 |
| *Rpl14* | Ribosomal protein L14 | structural component of ribosome | 1.34 | 1.30 | J0921H06 |
| *Dtna* | Dystrobrevin alpha | predicted in calcium/zinc regulation at the sarcolemma or synapse | 1.38 | 1.34 | H3105A04 |
| *Cyp51* | Cytochrome P450, family 51 | ergesterol biosynthesis | 1.48 | 1.55 | 0610008K05 |
| *Mxi1* | Max interacting protein 1 | transcriptional repressor, thought to negatively regulate myc | 1.50 | 1.50 | 1110038D23 |
| *Fn3k* | Fructosamine 3 kinase | fructosamine metabolism | 1.92 | 1.25 | 1810054D05 |
| **DEG in B-allele mice at 4 wk (4B) and 8 wk (8B)** | | | | | |
| **Gene Symbol** | **Gene Name** | **Function** | **4B Fold Change** | **8B Fold Change** | **Clone ID** |
| *Wnk2* | Wnk2, WNK lysine-deficient protein kinase 2 | Serine-threonine kinase | 1.21 | 1.32 | 1810073P09 |
| *Tbc1d23* | TBC1 domain family, member 23 | unknown | 1.26 | 1.40 | H3100C05 |
| *Aip* | Aryl-hydrocarbon receptor-interacting protein | unknown | 1.26 | 1.30 | UI-M-AO0-ace-g-03-0-UI |
| *Mmp2* | Matrix metalloproteinase 2 | calcium ion binding, collagen catabolism | 1.29 | 1.27 | 2310003M12 |
| *Scamp2* | Secretory carrier membrane protein 2 | recycling carrier to the cell surface | 1.32 | 1.45 | 0910001J19 |
| *Lrch1* | Lrch1, leucine-rich repeats and calponin homology (CH) domain containing 1 | contains gelsolin and leucine rich repeats | 1.33 | 1.29 | 1700014H06 |
| *Eif2s2* | eukaryotic translation initiation factor 2, subunit 2 | translation initiation | 1.35 | 1.36 | 1700027M17, 195785 |
| *Tep1* | Telomerase associated protein 1 | Telomerase protein component | 1.52 | 1.25 | H3142G05 |
| NA | NA | Unnamed | 1.22 | 1.44 | H3113A03 |
| **DEG in J-allele mice at 2 wk (2J) and 4 wk (4J)** | | | | | |
| **Gene Symbol** | **Gene Name** | **Function** | **2J Fold Change** | **4J Fold Change** | **Clone ID** |
| *Tbc1d23* | TBC1 domain family, member 23 | unknown | 1.64 | 1.20 | 1500002P11 |
| *Csf2* | Colony stimulating factor 2 (granulocyte-macrophage) | stimulates granulocyte and macrophage production | 1.46 | 1.25 | IMAGE:1445780 |
| **DEG in J-allele mice at 4 wk (4J) and 8 wk (8J)** | | | | | |
| **Gene Symbol** | **Gene Name** | **Function** | **4J Fold Change** | **8J Fold Change** | **Clone ID** |
| *Glud1* | Glutamate dehydrogenase 1 | synthesis and catabolism of glutamate | 0.74 | 0.76 | UI-M-AJ0-aaz-d-07-0-UI |
| *Plekha2* | Pleckstrin homology domain-containing, family A (phosphoinositide binding specific) member 2 | predicted to interact with PDZ-domain containing proteins | 0.76 | 0.70 | 1200009I02 |
| **DEG in B-allele mice at 2 wk (2B) and J-allele mice at 2 wk (2J)** | | | | | |
| **Gene Symbol** | **Gene Name** | **Function** | **2B Fold Change** | **2J Fold Change** | **Clone ID** |
| *Prdm4* | PR domain containing 4 | Transcription regulation | 1.41 | 1.38 | L0210F03 |
| *Hba-a1* | Hemoglobin alpha, adult chain 1 | Oxygen binding and transport | 1.67 | 1.42 | H3125H07 |
| *Zfp313* | Zinc finger protein 313 | Spermatogenesis, protein ubiquitination | 1.21 | 1.45 | 2610024O15 |
| *Pfkp* | Phosphofructokinase, platelet | phosphofructokinase activity, magnisium ion binding, glycolysis | 1.42 | 1.47 | 1200015H23 |
| NA | NA |  | 1.67 | 1.29 | 3200001C20 |
| **DEG in B-allele mice at 4 wk (4B) and J-allele mice at 4 wk (4J)** | | | | | |
| **Gene Symbol** | **Gene Name** | **Function** | **4B Fold Change** | **4J Fold Change** | **Clone ID** |
| *Tbc1d23* | TBC1 domain family, member 23 | unknown | 1.26 | 1.20 | J:1500002P11  B: H3100C05 |
| *Nvl* | Nuclear VCP-like | ATP-binding | 1.27 | 1.21 | 1200009I24 |
| *Rbm9* | RNA binding motif protein 9 | nuclear mRNA splicing via spliceosome | 1.30 | 1.26 | H3061C08 |
| NA | NA | Highly expressed in cerebellum | 1.32 | 1.21 | 1700022N22 |
| **DEG in B-allele mice at 8 wk (8B) and J-allele mice at 8 wk (8J)** | | | | | |
| **Gene Symbol** | **Gene Name** | **Function** | **8B Fold Change** | **8J Fold Change** | **Clone ID** |
| *Abt1* | Activator of basal transcription | General RNA polymerase II transcription factor activity | 1.24 | 1.21 | 2310031G24 |
| *Golph2* | Golgi phosphoprotein 2 | golgi protein of unknown function | 1.23 | 1.21 | 2810403H17 |
| *Rab8a* | Rab8a | DNA binding and regulation of transcription and the cell cycle | 1.59 | 1.25 | 2600001C15 |
| *Imp4* | IMP4, U3 small nucleolar ribonucleoprotein, homolog (yeast) | RNA binding; SnoRNP binding, rRNA processing | 1.28 | 1.26 | 1500019B21 |
| *Fubp3* | Far upstream element (FUSE) binding protein 3 | single-stranded DNA binding, transcriptional activator activity | 1.25 | 1.52 | 1110030O15 |
| *Lancl3* | LanC antibiotic synthetase component C-like 3 (bacterial) | unknown | 0.64 | 0.74 | 431294 |
| **Additional DEG in any B and any J** | | | | | |
| **Gene Symbol** | **Gene Name** | **Function** | **B Fold Change** | **J Fold Change** | **Clone ID** |
| *Ext* | Exostoses (multiple) 1 | transferring glycosyl groups; Golgi apparatus | 1.54 | 1.42 | 2310011A01 |
| *Dynll1* | Dynein, Light Chain, LC8 Type, 1 | microtubule motor activity | 1.48 | 1.51 | 200585 |
| *Cnnm2* | Cyclin M2 | unknown | 1.31 | 1.32 | H3086H12 |
| *Htatip2* | HIV-1 tat interactive protein 2, homolog (human) | Proapoptotic and antiapoptotic activitiy | 1.28 | 1.28 | 2200003B19 |
| *Smarcb1* | SWI/SNF related, matrix associated, actin dependent regulator of chromatin, subfamily b, member 1 | chromatin remodeling, regulation of transcription | 1.28 | 1.40 | 1810035N16 |
| *Cln6* | Ceroid-lipofuscinosis, neuronal 6 | lysosome organization and biogenesis | 1.27 | 1.46 | H3130G02 |
| *Bax* | Bcl2-associated X protein | caspase activation via cytochrome c | 1.27 | 1.24 | IMAGE:388733 |
| *Mmp2* | Matrix metallo-proteinase 2 | calcium ion binding, collagen catabolism | 1.27 | 1.35 | 2310003M12 |
| *Gdi1* | Guanosine diphosphate dissociation inhibitor 1 | GDP/GTP exchange | 1.25 | 1.41 | 1500001H20 |
| *Gadd45b* | Growth arrest and DNA-damage-inducible 45 β | activation of MAPKK, regulation of cell cycle | 1.24 | 1.69 | 2310034I12 |
| *Prdx4* | Peroxiredoxin 4 | activation of NFkappaB; peroxidase activity antioxidant activity | 1.23 | 1.61 | IMAGE:618400 |
| *Tusc3* | Tumor suppressor candidate 3 | unknown | 1.21 | 1.22 | 1500031L23 |
| *Gtdc1* | Glycosyltransferase-like domain containing 1 | unknown | 1.20 | 1.31 | H3091D07 |
| *Satb1* | Special AT-rich sequence binding protein 1 | maintenance of chromatin architecture, transcription factor activity | 1.20 | 1.56 | IMAGE:582944 |
| *Stmn2* | Stathmin-like 2 | growth cone, intracellular signaling | 0.80 | 0.67 | 2810404N08 |
| *Echdc1* | Enoyl Coenzyme A hydratase domain containing 1 | mitochondrial enzyme | 0.74 | 0.77 | H3085F11 |
| *Rem2* | Rad and gem related GTP binding protein 2 | DNA binding; regulation of transcription; GTP binding | 0.70 | 0.76 | 1190012L01 |
| NA | RIKEN cDNA 6330527O06 | Integral to membrane | 0.68 | 0.73 | 1110055G13 |
| NA | RIKEN full-length enriched library, clone: 6820436J05 | NA | 1.31 | 1.37 | H3149C07 |
| *Dcun1d5* | defective in cullin neddylation 1, domain containing 5 (S. cerevisiae) | unknown | 1.33 | 1.33 | 2610019N06 |
| NA | RIKEN cDNA 1810045K17 gene | NA | 1.41 | 1.24 | 1810049A01 |
| NA | CDNA BC004004 | NA | 1.42 | 1.20 | 2310033L20 |
| NA | NA | NA | 1.47 | 1.26 | IW:1251 |
| NA | NA | NA | 1.21 | 1.27 | 1810036L12 |
